# Therapeutic Effect of Repurposed Temsirolimus in Lung Adenocarcinoma Model

**DOI:** 10.3389/fphar.2018.00778

**Published:** 2018-07-24

**Authors:** Hsuen-Wen Chang, Min-Ju Wu, Zih-Miao Lin, Chueh-Yi Wang, Shu-Yun Cheng, Yen-Kuang Lin, Yen-Hung Chow, Hui-Ju Ch’ang, Vincent H. S. Chang

**Affiliations:** ^1^Laboratory Animal Center, Office of Research and Development, Taipei Medical University, Taipei, Taiwan; ^2^The PhD Program for Translational Medicine, College of Medical Science and Technology, Taipei Medical University, Taipei, Taiwan; ^3^Biostatistics Research Center, Taipei Medical University, Taipei, Taiwan; ^4^National Institutes of Infectious Diseases and Vaccinology, National Health Research Institutes, Zhunan, Taiwan; ^5^National Institute of Cancer Research, National Health Research Institutes, Zhunan, Taiwan

**Keywords:** mTOR inhibitor, drug repositioning, temsirolimus, lung adenocarcinoma, chemotherapy

## Abstract

Lung cancer is one of the major cause of cancer-related deaths worldwide. The poor prognosis and resistance to both radiation and chemotherapy urged the development of potential targets for lung cancer treatment. In this study, using a network-based cellular signature bioinformatics approach, we repurposed a clinically approved mTOR inhibitor for renal cell carcinomans, temsirolimus, as the potential therapeutic candidate for lung adenocarcinoma. The PI3K-AKT-mTOR pathway is known as one of the most frequently dysregulated pathway in cancers, including non-small-cell lung cancer. By using a well-documented lung adenocarcinoma mouse model of human pathophysiology, we examined the effect of temsirolimus on the growth of lung adenocarcinoma *in vitro* and *in vivo*. In addition, temsirolimus combined with reduced doses of cisplatin and gemcitabine significantly inhibited the lung tumor growth in the lung adenocarcinoma mouse model compared with the temsirolimus alone or the conventional cisplatin–gemcitabine combination. Functional imaging techniques and microscopic analyses were used to reveal the response mechanisms. Extensive immunohistochemical analyses were used to demonstrate the apparent effects of combined treatments on tumor architecture, vasculature, apoptosis, and the mTOR-pathway. The present findings urge the further exploration of temsirolimus in combination with chemotherapy for treating lung adenocarcinoma.

## Introduction

Lung cancer is one of the most common forms of cancer and remains the number one cause of cancer-related deaths worldwide among men and women. Based on histological differentiation, there are two major types of lung cancers: small-cell lung cancer (SCLC) and non-small-cell lung cancer (NSCLC). NSCLCs are further divided into squamous cell carcinomas (SCCs), pulmonary adenocarcinomas (ADC), and large-cell carcinomas. Among them, lung ADC is the most prevalent form of NSCLC (Teng, 2005; Chang et al., 2017). Lung cancer has a dismal prognosis of 15%, mainly attributed to ineffective early detection and lack of therapeutic options for metastatic disease (Molina et al., 2008). This has spurred efforts for the development of molecularly targeted therapies.

The definition of drug repositioning is to identify new indications from existing drugs or compounds to treat a different disease. In addition to being time- and cost-efficient, drug repositioning offers a more favorable risk-versus-reward trade-off of the available drug development strategies. Because the existing drugs have already been tested in terms of safety, dosage, and toxicity, they can often enter clinical trials much more rapidly than newly developed drugs (Ashburn and Thor, 2004). Computational drug repositioning is deemed as an alternative and effective way to identifying novel connections between diseases and existing drugs (Hurle et al., 2013). The increase in drug-target information and advances in systems pharmacology approaches have led to an increase in the success of *in silico* drug repositioning. In particular, large-scale genomics databases, such as the Connectivity Map, provide abundant information on the modes of action of drugs, which are reflected in the transcriptomic responses to chemical perturbation (Vempati et al., 2014). Recently, a similar but highly expanded version of a chemical genomics dataset was publicly released by the National Institutes of Health Library of Integrated Network-Based Cellular Signatures (NIH LINCS) program. This dataset includes gene expression signatures and protein binding, cellular phenotypic, and phosphoproteomics profiles resulted from chemical or genetic perturbation. Specifically, it presents the gene expression profiles of approximately 1000 landmark genes (L1000) in response to more than 20,000 chemical perturbations across many cell lines. Additionally, transcriptome-level expression profiles of approximately 20,000 genes have been computationally inferred using 1000 landmark genes (Vempati et al., 2014).

In this study, we compared the transcriptome profiles obtained from a well-documented mouse lung cancer model (Chang et al., 2017) and used the LINCS L1000 cellular signature bioinformatics approach to identify clinically approved candidate drugs to treat ADC. By using this strategy, we identified temsirolimus, a mTOR inhibitor approved for renal cell carcinoma, as a potential therapeutic agent for the treatment of lung tumor. In a study using mouse model xenografted with human NSCLC cells (A549, H1299, and H358), it was found that temsirolimus could inhibit the growth of subcutaneous tumors, as well as to prolong the survival of mice having pleural dissemination of cancer cells due to its anti-proliferative effect (Ohara et al., 2011). Temsirolimus also has been used on a case report (Vichai and Kirtikara, 2006) with lung adenocarcinoma harboring specific gene mutation; it was also noted to restore radio-sensitivity in lung adenocarcinoma cell lines (Ushijima et al., 2015). Two updated phase two clinical trials of temsirolimus (Study 1: Neratinib with and without temsirolimus for patients with HER2 activating mutations in non-small cell lung cancer. Study 2: Temsirolimus and pemetrexed for recurrent or refractory non-small cell lung cancer.) were found from webpage searching^1^, either as monotherapy or combined therapy with another drug. Although there are more than 40 inhibitors of the PI3K-AKT-mTOR signaling pathway have reached different stages of clinical development, only a few have been approved for clinical use (Skehan et al., 1990). However, an *in vivo* systemic evaluation of the lung tumor inhibitory effect of temsirolimus was lack. Here we assessed the combination of the mTOR inhibitor temsirolimus with the first-line chemotherapy for advanced NSCLC, cisplatin, and gemcitabine, to reduce cytotoxicity and enhance the therapeutic response.

## Materials and Methods

### Microarray Analysis

Total RNA was extracted from tissue samples or cells by using TRIzol^®^ Reagent (Sigma, St. Louis, MO, United States) by following the manufacturer’s instructions. Total RNA (0.2 μg) was amplified as previously mentioned (Chang et al., 2017) for microarray analysis by using a microarray scanner (Agilent Technologies, Santa Clara, CA, United States). A total of 155 differentially expressed genes were identified in the Tg-3m mice, of which 126 genes were upregulated (a log2 fold change of ≥0.6) and 29 genes were downregulated (a log2 fold change of ≤−0.6). A total of 123 differentially expressed genes were identified in the Tg-6m mice, of which 105 genes were upregulated (a log2 fold change of ≥0.6) and 18 genes were downregulated (a log2 fold change of ≤ −0.6).

### LINCS Perturbagen Signature Comparisons

The LINCS L1000 is one of the complete drug treatment expression profile databases and currently contains more than a million gene expression profiles of chemically perturbed human cell lines (Blois et al., 2011; Li et al., 2014). First, for comparing the gene expression signatures in transgenic mice with LINCS data sets, the gene expression data were ranked according to the log2 fold changes. We retrieved the top 100 and bottom 100 most differentially expressed genes as gene expression signatures in both Tg-3m and Tg-6m mice. Then, we transferred the mouse gene symbols to homologous human gene symbols by using the HomoloGene database (Gabriel et al., 2016). Next, we queried the homologous human genes against the LINCS database by using sig_query and sig_summly in the LINCS C3 server. Finally, we annotated the returned results by combining the DrugBank (Vichai and Kirtikara, 2006) and PubChem (Ushijima et al., 2015) results to provide detailed perturbagen information.

### Functional Annotation of Differentially Expressed Genes

To discuss the gene ontology and Kyoto Encyclopedia of Genes and Genomes pathways involved in transgenic mice, we analyzed the differentially expressed genes by using the Database for Annotation, Visualization and Integrated Discovery (DAVID, version 6.7 ^2^) (Huang da et al., 2009) application programming interfaces (APIs). A *p*-value of 0.05 was set as the threshold, which was calculated using Fisher’s exact test.

### Animals and Ethics Statement

Murine lung adenocarcinoma models were maintained as previously mentioned (Chang et al., 2017) in a specific pathogen-free environment at the animal facility of Taipei Medical University. Experimental uses of mice were approved by the Institutional Animal Care and Use Committee of Taipei Medical University (Approved Proposal No. LAC-2014-0217). All experiments were conducted in accordance with relevant guidelines and regulations. The mice were monitored daily for physiological conditions. Tumor growths were monitored using micro-CT on a weekly basis. Mice were anesthetized by administering 5% isoflurane followed by 2% isoflurane through the inhalation route for maintenance during the imaging process. Total lung volumes were measured and analyzed using CTAn software (v.1.15), and mice were euthanized when the total lung volumes were less than 120 mm^3^. At the endpoint of the experiment (the 16th week), the tested mice were euthanized by administering 100% CO_2_ through inhalation to minimize their suffering.

### Cell Cycle and Apoptosis Assays

The effects of temsirolimus and chemotherapy on the cell cycle and apoptosis were evaluated by seeding tumor cells into 6-well plates at a density of 5 × 10^4^ per well. The cells were treated accordingly and incubated for 24 h followed by a phosphate-buffered saline (PBS) wash. The cell cycle phases were determined using a Muse cell analyzer (Merck Millipore, Darmstadt, Germany) and a Muse Cell Cycle Assay Kit (Merck Millipore, Darmstadt, Germany) according to the manufacturer’s instructions. Cell apoptosis was analyzed using Annexin V Dead cell reagent (Merck Millipore, Darmstadt, Germany) according to the manufacturer’s instructions. An average of at least 10,000 cells was analyzed for each condition. Triplicate independent experiments were conducted.

### Protein Preparation and Western Blotting

Protein extraction and Western blotting analysis were performed as previously mentioned (Chang et al., 2017). The blots were immunostained with 1:1000 of anti-p-mTOR (Ser2448) antibody (2971, Cell Signaling, Danvers, MA, United States). After incubation with horseradish peroxidase-conjugated secondary antibody (1:4000 of goat antirabbit IgG, GTX213110-01, GeneTex, Irvine, CA, United States), protein bands were visualized with an enhanced chemiluminescent reagent.

### Micro-CT

Mice were anesthetized with an induction flow dose of 3% isoflurane and oxygen mixture, following a maintaining flow dose of 1%. The chest area was scanned at one time through *in vivo* micro-CT (Bruker SkyScan 1176, Kontich, Belgium). Image scanning was performed in resolution of 35 μm. The instrument setting was at a voltage of 50 kVp, a current of 500 μA, and an exposure time of 50 ms with a 0.5-mm aluminum filter. To prevent artifacts caused by cardiac and respiratory motion, images were captured using the synchronization mode. Sections were reconstructed using a graphics processing unit-based NRecon software. The tumor volume inside the lung area was separated and analyzed using CTAn software (Bruker SkyScan, Kontich, Belgium). The cross-sectional images were obtained using DataViewer software (Bruker Skyscan, Kontich, Belgium).

### Histology and Immunohistochemistry

Mouse lung tumors were removed and prepared for paraffin-embedded sectioning immunohistochemistry (IHC) staining was performed as previously mentioned (Chang et al., 2017). After antigen retrieval, primary antibody dilutions were prepared in the blocking buffer (10% bovine serum albumin with 0.1% Triton-100 in PBS) as follows: 1:200 of anti-Ki67 antibody (ab15580, Abcam, Cambridge, MA, United States), 1:250 of anti-CD34 antibody (ab81289, Abcam, Cambridge, MA, United States), 1:100 of p-mTOR (ab109268, Abcam, Cambridge, MA, United States), and 1:400 of p-S6RP antibody (2211, Cell Signaling, Danvers, MA, United States). Immunochemical signals were detected using a MultiLink Detection Kit (BioGenex, Fremont, CA, United States). The peroxidase reaction was developed with diaminobenzidine, and sections were counterstained using Mayer’s hematoxylin. The intensity of positive signal areas was measured using ImageJ software (IJ 1.46r).

### Statistical Analyses

SAS version 9.3 for Windows (SAS Institute, Cary, NC, United States) was used for data manipulation and visualization. The means are used to describe the central tendency of continuous variables while standard deviations are used to depict the variation. One-way ANOVA and the Bonferroni *post hoc* multiple comparison tests were of inhibitory effects among different treatments. All statistical analyses were two sided, and *p* < 0.05 was considered as statistically significant. *p*-Values were depicted using asterisks, with ^∗^*p* < 0.05, ^∗∗^*p* < 0.01.

## Results

### Data Processing and Drug Repositioning

**FIGURE 1:**
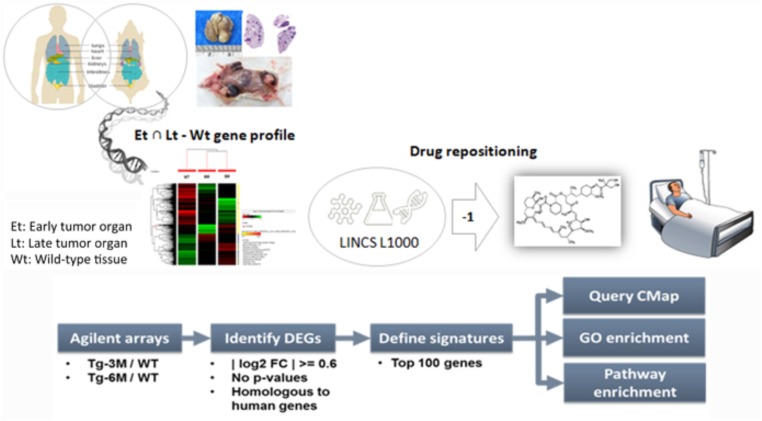
Bioinformatics-based drug-repositioning approach to identify candidate drugs. Schematic representation of the bioinformatics workflow by using the LINCS L1000 data set for the repositioning approach to identifying potential candidate drugs for the treatment of NSCLC. The microarray results of Tg-3m and Tg-6m tumors were subjected to the LINCS L1000 data sets to obtain the most differentially expressed genes. The mouse gene symbols were then converted to human homologous genes and annotated by combining the DrugBank and PubChem results to obtain detailed drug information. The drugs that negatively (K score = –1) correlated with the gene expression from both Tg-3m and Tg-6m lung tumor cell lines were selected for further screening.

To compare the gene expression signatures from different stages of lung tumors, microarray results of Tg-3m and Tg-6m tumors (Chang et al., 2017) were subjected to the LINCS L1000 data sets, and the gene expression data were ranked according to the log2 fold changes. The top 100 and bottom 100 most differentially expressed genes were retrieved as gene expression signatures in both Tg-3m and Tg-6m mice. The mouse gene symbols were then converted to homologous human gene symbols by using the HomoloGene database (Coordinators, 2016). Next, the homologous human genes were queried against the LINCS database by using sig_query and sig_summly in the LINCS C3 server. The returned results were annotated by combining the DrugBank (Wishart et al., 2006) and PubChem (Kim et al., 2016) results to obtain detailed drug information (**Figure 1**). The drugs that negatively (K score = −1) correlated with the gene expression from both Tg-3m and Tg-6m lung tumor cell lines were selected for further screening. The data regarding the drugs were then manually curated from DrugBank and PubMed by searching for keywords and abstracts that explicitly described their association with cancers. The repositioned drug candidates are listed in **Table 1**. This list contained a wide range of drugs, including some antineoplastic agents used for cancers other than lung cancer, suggesting that the use of these agents in clinics may affect the gene expression signature of lung cancer (Kerr et al., 2007; Lin et al., 2007; Gallotta et al., 2010; Wynne and Djakiew, 2010; Endo et al., 2014; Li et al., 2016). We focused on the top-scoring candidates and clinically approved antineoplastic drugs. This analysis led to the identification of temsirolimus, a U.S. Food and Drug Administration (FDA)-approved mTOR inhibitor for renal cell carcinoma, which was repositioned from both stages of lung tumor cells and was tested in combination with thoracic radiation in NSCLC (Waqar et al., 2014).

**Table 1:** List of drug repositioning candidates.

Drug repositioning from Tg-3m tumor cells			
Name	K score	Original indication	Cancer indication/clinical trials^+^
Mesoridazine	−1	Antipsychotic	N/A
Dexamethasone	−1	Anti-inflammatory; steroids	Myeloma
Nilotinib	−1	Antineoplastic	Leukemia
Testosterone	−1	Anabolic	Prostate cancer
**Temsirolimus**	−0.99	Antineoplastic	Renal cell carcinoma
Prazosin	−0.98	Adrenergic	Prostate cancer
Pipamperone	−0.95	Antipsychotic	N/A
Rifabutin	−0.94	Antibiotic	Lung cancer
Omeprazole	−0.94	Anti-ulcer	Head and neck cancer
Cytarabine	−0.94	Antineoplastic	Leukemia
Timolol	−0.94	Adrenergic	N/A
Rofecoxib	−0.94	Analgesics	Colorectal cancer
Ibuprofen	−0.94	Analgesics	Lung cancer; prostate cancer
Ranitidine	−0.94	Anti-ulcer	Myeloma, renal cell carcinoma
**Drug repositioning from Tg-6m tumor cells**			
Triamcinolone	−1	Anti-inflammatory; steroids	N/A
Flurbiprofen	−0.98	Analgesics	Prostate cancer
Rimonabant	−0.98	Antiobesity	Leukemia
Tamoxifen	−0.98	Anti-estrogen; antineoplastic	Breast cancer
**Temsirolimus**	−0.98	Antineoplastic	Renal cell carcinoma
Nicorandil	−0.98	Vasodialator	N/A

*^+^Information obtained from DrugBank (https://www.drugbank.ca/).*

### Temsirolimus Treatment Leads to G_0_/G_1_ Cell Cycle Arrest

**FIGURE 2:**
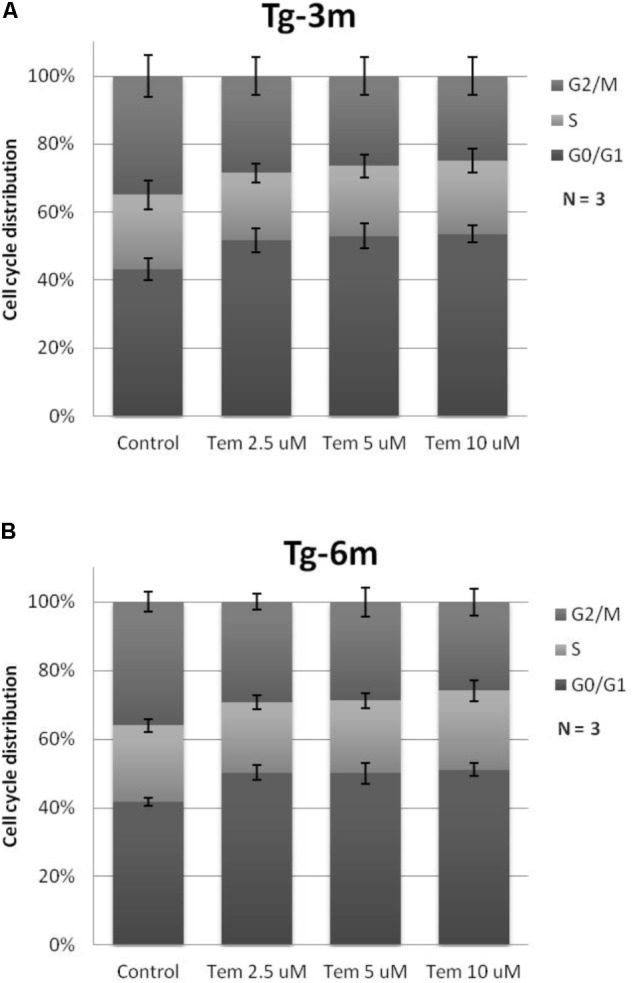
Cytostatic effect caused by temsirolimus at different concentrations in both Tg-3m **(A)** and Tg-6m **(B)** lung tumor cell lines. Temsirolimus treatment resulted in the cell arrest at the G1 phase in a concentration-dependent manner. The representative data showed the results from three independent experiment.

**FIGURE 3:**
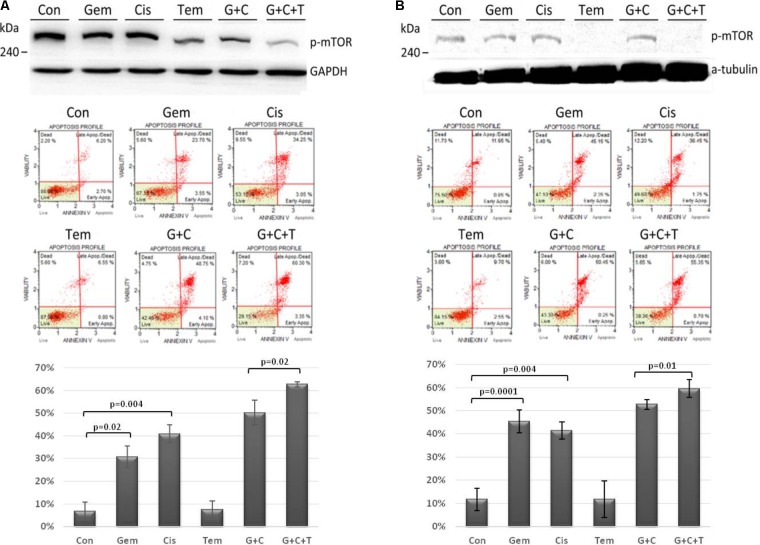
Temsirolimus combined with cisplatin and gemcitabine induced significant apoptotic cell death through the inhibition of p-mTOR in both Tg-3m **(A)** and Tg-6m **(B)** lung tumor cell lines. Although temsirolimus treatment alone did not cause cell apoptosis, when combined with cisplatin and gemcitabine, it significantly enhanced the cytotoxicity by approximately 10% in Tg-3m and Tg-6m cells (*p* = 0.02 and 0.01, respectively), which was higher than that caused by the doublet of cisplatin and gemcitabine. Either gemcitabine or cisplatin alone also showed statistical significance from control. Con: control, Gem: gemcitabine, Cis: cisplatin, Tem: temsirolimus, G + C: gemcitabine + cisplatin, G + C + T: gemcitabine + cisplatin + temsirolimus.

To understand whether temsirolimus treatment is lethal to lung tumor cells at both early and late stages, we performed flow cytometry to analyze the cell cycle distribution in Tg-3m (**Figure 2A**) and Tg-6m (**Figure 2B**) cell lines treated with temsirolimus at different concentrations (2.5, 5.0, and 10 μM). Temsirolimus treatment increased the cell population in the G_0_/G_1_ phase in both Tg-3m and Tg-6m cell lines but did not cause significant cell death (**Figure 3**). Taken together, these results suggest that temsirolimus suppressed the proliferation of Tg-3m and Tg-6m cells through its cytostatic effect and not through cytotoxicity.

### Efficacy of Temsirolimus, Cisplatin, and Gemcitabine in mTOR Pathway and Cytotoxicity

The efficacy of temsirolimus, cisplatin, and gemcitabine (each at 10 μM) alone and in combination was evaluated in Tg-3m (**Figure 3A**) and Tg-6m (**Figure 3B**) cells. To evaluate the effect of temsirolimus on activation regulation in the mTOR pathway, we examined the phosphorylation of mTOR (s2448) by using Western blot analysis. Gemcitabine or cisplatin treatment did not alter the phosphorylation of mTOR. However, treatment with temsirolimus alone markedly suppressed the activation of mTOR in Tg-6m than in Tg-3m cells. When cells were treated with temsirolimus combined with cisplatin and gemcitabine, the effect of mTOR suppression was evident. The apoptotic cell death in H1299 human NSCLC cell line was presented in **Supplementary Figure S3**.

To evaluate the cytotoxic effect of temsirolimus, we examined the total cell apoptotic rate by using annexin V staining. In human NSCLC cell line H1299 treated with temsirolimus alone caused about 25% cell death, when combined with cisplatin and gemcitabine showed enhanced cytotoxicity by approximately 10% in G + C and 15% in G + C + T (*p* = 0.02 and 0.003, respectively) (**Supplementary Figure S4**). Treatment with gemcitabine alone induces higher cytotoxicity in Tg-6m than in Tg-3m cells; however, treatment with cisplatin alone did not reveal any substantial difference. Treatment with gemcitabine plus cisplatin revealed similar apoptotic results in both cell lines. Although treatment with temsirolimus alone did not cause cytotoxicity, it enhanced the cisplatin and gemcitabine-induced apoptosis in both cell lines significantly (*p* < 0.05; **Figures 3A,B**).

### Treatment Effects of Temsirolimus, Cisplatin, and Gemcitabine on Tumor Growth

**FIGURE 4:**
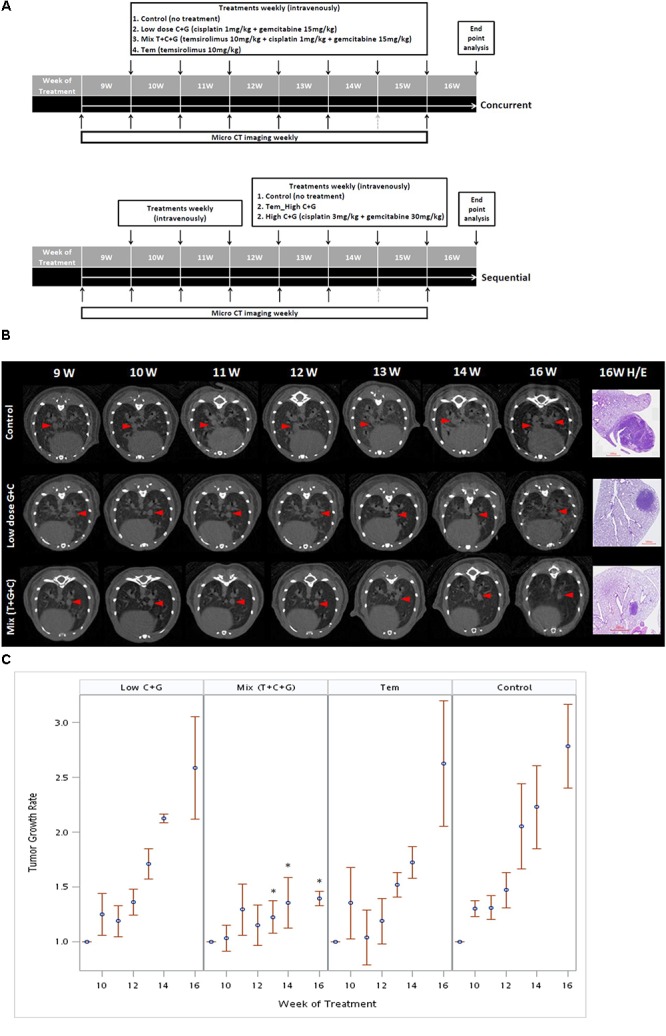
Treatment sequences of temsirolimus alone (Tem), low-dose chemotherapy (Low C + G) and temsirolimus combined with low-dose chemotherapy (Mix T + C + G) in the lung tumor mouse model. Mice were treated at the age of 9 weeks for 7 weeks. The lung tumor growths were monitored using micro-CT every week, except for the 15th week because of regular maintenance of the scanner (gray-dotted arrow). Concurrent and sequential administration of treatments were depicted **(A)**. Red arrow heads indicate the monitored tumors compared with the corresponding H&E-stained histopathologic sections at the endpoint **(B)**. The endpoint H&E-stained sectioned sections were also displayed as inset in **Figure 5**. The tumor growth rate was calculated by normalizing each tumor volume to the baseline tumor volume of each mouse at the beginning of week 9. The effect of different treatments: Tem, Low C + G and Mix T + C + G in lung tumor growth inhibition in time periods were displayed **(C)**. The significance of tumor growth inhibition among each treatment was analyzed and it was found significant after week 13 in the Mix (T + C + G) group compared to the control group (^∗^*p* ≤ 0.05).

To investigate the effect of temsirolimus, cisplatin, and gemcitabine on tumor growth, we used a therapeutic approach with a previously documented NSCLC mouse model (Chang et al., 2017). The mice were divided into three groups (*n* = 5): the control group (no treatment), the group that received a low dosage of cisplatin and gemcitabine (low-dose C + G), and the group that received temsirolimus combined with a low dosage of cisplatin and gemcitabine (mix T + C + G). The mice were treated at the age of 9 weeks for 8 weeks. Both treatments were administered weekly through the tail vein, and micro-CT imaging was performed to follow up tumor growths (**Figure 4A**). The imaging on week 15 was postponed because of regular maintenance of the scanner. On week 16, the mice were sacrificed and their lungs were removed for histopathological analysis.

The tumor growth rate was calculated by normalizing each tumor volume to the baseline tumor volume of each mouse at the beginning of week 9. The tumor growth was slightly reduced in the low C + G group, whereas it was markedly inhibited in the mix (T + C + G) group. In addition, the tumor growth significantly declined after 4 weeks’ treatment in the mix (T + C + G) group with *p* ≤ 0.05 (weeks 13–16). Smaller and reduced lung tumors were also noted in hematoxylin and eosin (H&E)-stained lung sections (**Figures 4B,C**). Collectively, the weekly administration of temsirolimus combined with low doses of cisplatin and gemcitabine effectively reduced the growth of lung tumors.

### Treatment Effects on General Tumor Characteristics and the mTOR-Pathway

**FIGURE 5:**
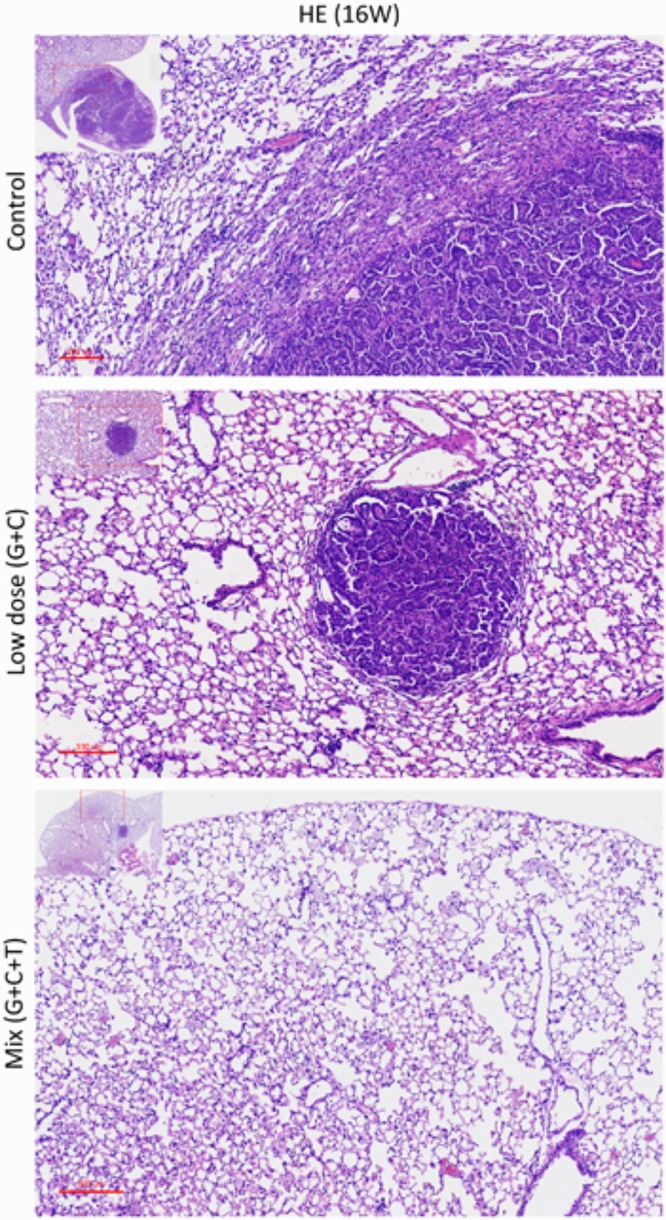
Histopathologic views of treatment effects. Representative sections of the lung tissue of mice after various treatments. Viable tumor mass within the lung parenchyma with immune cell infiltration (control). Residual tumor mass within the lung parenchyma with congestion, hyaline deposition, and immune cell infiltration (low-dose C + G). Scattered tumor cells with nuclear pleomorphism within the lung parenchyma with congestion, foamy macrophages, and giant cells (mix T + C + G). H&E-stained slides of sections from mice with lung tumors were assessed by pathologists blinded to the treatment and outcome. Magnification: 100× and 40× (inset).

**FIGURE 6:**
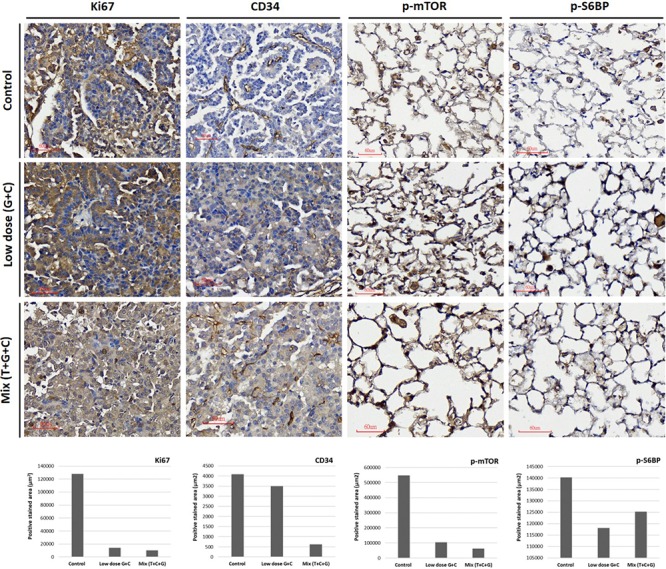
Treatment effects on tumor proliferation, angiogenesis, and the mTOR-pathway. Representative images of Ki67, CD34, p-mTOR, and p-S6RP expression were immunostained using specific antibodies as indicated. Tumor proliferative signal of Ki67 was more condensed in untreated tumors and more diffuse in low-dose and mixed treatment groups. The angiogenetic architecture was more intact in untreated tumors compared with treated groups, as analyzed using CD34 staining. The inhibition of p-mTOR expression was higher in the mixed treatment groups. The phosphorylation of S6RP was also examined as a downstream target of the mTOR-pathway. The p-S6RP expression was reduced after both treatments. Image magnification: 300× in Ki67 and 400× in CD34, p-mTOR, and p-S6RP. Quantitative analysis of Ki67, CD34, p-mTOR, and p-S6RP expression in IHC-stained section were analyzed and present. The whole positive stained areas (μm^2^) of each representative image were measured using ImageJ software and visualized as bar chart below.

At the end of the experiment (week 16), all lungs were dissected and immunohistochemically analyzed to assess and quantify the microscopic effects of combined therapies with or without temsirolimus on general tumor characteristics (H&E stain; Ki-67 and CD34) and to identify possible mechanisms for the observed differences in growth inhibition. H&E staining revealed viable tumor mass within the lung parenchyma in untreated tumors, with immune cell infiltration. Residual tumor mass within the lung parenchyma with congestion, hyaline deposition, and immune cell infiltration were observed in low-dose C + G treated tumors. Scattered viable tumor cells with nuclear pleomorphism within the lung parenchyma revealed foamy macrophages and giant cells when treated with combined T + C + G after chemotherapy (magnification: 100×; **Figure 5**). Ki-67 staining revealed condensed signals of proliferating tumor cells in untreated control tumors. Treatment with low-dose C + G resulted in a lower fraction of proliferating cells, whereas that with combined T + C + G demonstrated diffused proliferating signals (magnification: 300×). CD34 staining demonstrated disruptive angiogenetic architectures in the low-dose and mix groups compared with the untreated control groups (magnification: 400×). In addition to general tumor characteristics, we investigated specific treatment effects on the mTOR pathway by evaluating p-mTOR and pS6RP in all lung tumors (magnification: 400×; **Figure 6**). The quantitative bar charts represent the positively stained areas of the whole image above, revealed that both treatments inhibited the tumor proliferation marker of Ki67. The combination treatment with temsirolimus markedly inhibited angiogenesis compared with low-dose chemotherapy. Quantitative stained areas demonstrated reduced p-mTOR signaling in both the treated groups, whereas the p-S6BP signal was higher. The statistical analysis of the intensity of positive signals from three selected views of each IHC-stained section demonstrated similar results (**Supplementary Figure S5**). Whether the p-S6BP signaling resulted from heterogeneous tumor cells remains to be investigated.

## Discussion

Chemotherapy is one of the most important treatment methods for advanced NSCLC, and cisplatin-based combinations are usually used as standard regimens. The combination of one or more agents with a platinum compound resulted in high response rates and prolonged survival (Schiller et al., 2002; Ruiz-Ceja and Chirino, 2017). Gemcitabine was approved by FDA in 1996 with DNA synthesis inhibition. Gemcitabine is indicated in combination with cisplatin as the first-line treatment of patients with advanced NSCLC (Ruiz-Ceja and Chirino, 2017). Common cisplatin plus gemcitabine treatment-related adverse events are hematologic toxicity and gastrointestinal reaction. Hematologic toxicity mainly included decreased white blood cells and platelets. Gastrointestinal reactions mainly included nausea and vomiting (Ai et al., 2016). However, the high toxicity induced by cisplatin-based doublets urges research on alternative treatments. In this study, we used the LINCS L1000 database and a well-characterized lung adenocarcinoma mouse model to repurpose existing drugs for lung adenocarcinoma. By using this approach, we identified the mTOR inhibitor, temsirolimus, which has been approved by the FDA for renal cell carcinoma, as a potential therapeutic agent. In our results, both temsirolimus-treated early (Tg-3m) and late-stage (Tg-6m) lung tumor cell lines demonstrated cell cycle arrest at the G0/G1 phase. The treatment with temsirolimus alone markedly suppressed mTOR activation in Tg-6m than in Tg-3m cells. When temsirolimus was combined with cisplatin and gemcitabine, the effect of mTOR suppression was evident. Additionally, temsirolimus combined with gemcitabine and cisplatin not only suppressed the phosphorylation of mTOR but also significantly improved cell death in Tg-3m and Tg-6m cell lines compared with gemcitabine plus cisplatin.

As reported by Khuri colleagues (Li et al., 2014), mTOR inhibition triggers rapid and sustained activation of the PI3K/Akt survival pathway in the human lung and other types of cancer cells; therefore, the combination of mTOR-targeted therapy with drugs that block PI3K/Akt activation might also be reasonable. In a reported phase II study, temsirolimus was administered as a single agent in 52 patients with untreated NSCLC on a weekly basis. The clinical benefit rate was 35%, with a confirmed partial response of 8% and stable disease of 27%. Although these results did not satisfy the protocol-defined criteria for success, they evidenced the clinical activity of temsirolimus as a single agent in NSCLC (Reungwetwattana et al., 2012). In a phase I study, temsirolimus was combined with weekly thoracic radiation, which proved the tolerance (Waqar et al., 2014). Because temsirolimus has demonstrated considerable activity in clinical studies, we hypothesize that it works synergistically with the first-line NSCLC chemotherapy cisplatin plus gemcitabine.

In animal studies, optimizing the cytostatic agent temsirolimus with cycle-active chemotherapy is important for maximizing the clinical benefit. Therefore, we designed concurrent and sequential administration of temsirolimus with either low or high doses of chemotherapy in a mouse lung adenocarcinoma model. In the concurrent schedule, administration of low-dose chemotherapy and temsirolimus (T + C + G) demonstrated greater inhibition of tumor growth compared with low-dose chemotherapy alone (C + G) in the mouse model. In the sequential schedule in which temsirolimus alone was administrated weekly for 3 weeks prior to the administration of high-dose chemotherapy (3 mg/kg cisplatin + 30 mg/kg gemcitabine) in the following weeks, the effect of tumor growth inhibition was less significant (**Supplementary Figures S1**, **S2**). Collectively, our study revealed that concurrent administration of low-dose chemotherapy and temsirolimus is more effective in suppressing lung tumor growth, which may be advantageous to reduce the cytotoxicity caused by standard chemotherapy. The histopathologic evaluation of endpoint H&E-stained lung tumor sections revealed that the tumors were associated with an extensive response to the T + C + G treatment compared with low-dose C + G treatment. Common tumorigenic and angiogenetic markers (Ki67 and CD34) were apparently inhibited after the T + C + G treatment compared with low-dose C + G treatment. These results proved the tumor inhibition efficacy of temsirolimus combined with low-dose chemotherapy. The mTOR phosphorylation inhibition was higher in the mixed treatment. Moreover, the phosphorylation of the ribosomal protein S6 (p-S6RP), one of the targets downstream of the mTOR pathway, was reduced after both treatments. The examination of phosphorylated mTOR and S6RP suggested their sensitivity to temsirolimus.

The clinical benefits of chemotherapy are limited by drug resistance and systemic toxicity. Temsirolimus was reported to restore cisplatin sensitivity in lung cancer cell lines by blocking the translation of proteins that are involved in cisplatin resistance (Blois et al., 2011). The cytostatic effect of temsirolimus was also demonstrated by introducing temsirolimus as a molecular-targeted agent with the potential for inhibiting tumor cell repopulation (Fung et al., 2009). However, the pulmonary toxicity was associated with mTOR inhibitors as many other drugs, including anticancer agents (Blois et al., 2011; Li et al., 2014). Proper chemotherapeutic strategy management and clinical pulmonary symptom diagnosis should be taken account when administration with mTOR inhibitors. Our study demonstrated that a combination of low-dose chemotherapy and temsirolimus treatment was more effective in inhibiting tumor growth than a doublet chemotherapy regimen in the mouse lung tumor model. In addition, the concurrent administration of the combined treatment was more efficacious than the sequential administration of these agents at a higher dose. Our study results suggest that the combination of low-dose chemotherapy and temsirolimus treatment might be beneficial in the treatment of lung adenocarcinoma, which warrants further investigation.

## Author Contributions

H-WC and VC conceived the experiments. M-JW and Z-ML conducted the experiments. C-YW conducted the micro-CT imaging. S-YC conducted the tissue embedding and histopathology. H-WC, H-JC, and VC analyzed the results. Y-KL assisted on statistical analysis. H-WC wrote up the manuscript. Y-HC and VC provided comments on the manuscript. All authors reviewed the manuscript.

## Conflict of Interest Statement

The authors declare that the research was conducted in the absence of any commercial or financial relationships that could be construed as a potential conflict of interest.

## Footnotes

1 https://clinicaltrials.gov/beta/

2 david.ncifcrf.gov

## Supplementary Material

FIGURE S1 Effects of high-dose chemotherapy and the sequential administration of temsirolimus followed by high-dose chemotherapy in lung tumor growth inhibition. The tumor growth inhibition was significant in the first 3 weeks of treatment with temsirolimus. However, the tumor growth inhibition efficacies between the two chemotherapy regimens were similar **(A)**. Effect of various treatments on tumor growth rate **(B)**. The tumor growth inhibition was most significant after the mixed treatment (T + C + G) with a lower dose of chemotherapy, which is beneficial in reducing the cytotoxic effect. (^∗^*p* ≤ 0.05; ^∗∗^*p* ≤ 0.01).

FIGURE S2 Effect of temsirolimus alone (orange line) compared to different regimes of chemotherapy in lung tumor growth inhibition. The tumor growth inhibition of different treatments was depicted with statistical significance. While the tumor growth was inhibited moderately using temsirolimus alone (orange line), combined temsirolimus with low-dose chemotherapy (blue line, *p*-value = 0.019) inhibited the tumor growth significantly. Combined temsirolimus with low-dose chemotherapy treatment also showed significance over the treatment using temsirolimus alone or high-dose chemotherapy (black line), with *p*-value = 0.037 and 0.032, respectively.

FIGURE S3 Temsirolimus combined with cisplatin and gemcitabine induced apoptotic cell death in H1299 human NSCLC cell line. Although temsirolimus treatment alone caused about 25% cell death, when combined with cisplatin and gemcitabine, it significantly enhanced the cytotoxicity by approximately 10% in G + C and 15% in G + C + T (*p*-value = 0.02 and 0.003, respectively). Con: control, Gem: gemcitabine, Cis: cisplatin, Tem: temsirolimus, G + C: gemcitabine+cisplatin, G + C + T: gemcitabine + cisplatin + temsirolimus.

FIGURE S4 SRB cytotoxicity assay performed in Tg-3m **(A)** and Tg-6m **(B)** cell lines. The results showed either temsirolimus alone or combined with cisplatin and gemcitabine displayed significant cytotoxic effects in both Tg-3m and Tg-6m cell lines. The results of SRB assay demonstrated the *in vitro* correlation of cytotoxicity and cell death caused by chemotherapeutic agents tested.

FIGURE S5 Statistical quantification of IHC-stained sections of Ki67, CD37, p-mTOR, and p-S6RP. The intensity of positive signals from each IHC-stained section were selected from three different views (red-lined squares) and analyzed using Image J software with IHC toolbox as plugins.
