# Supplementary figures and images for: Therapeutic Effect of Repurposed Temsirolimus in Lung Adenocarcinoma Model
Source: Front Pharmacol. 2018 Jul 24;9:778. doi: 10.3389/fphar.2018.00778 (PMC6066584; doi:10.3389/fphar.2018.00778)

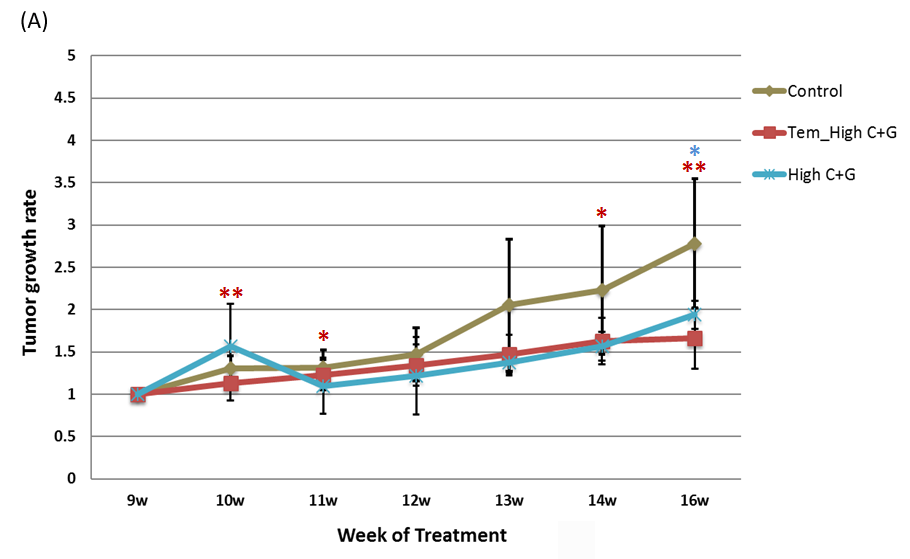

Supplement: FIGURE S1 — Effects of high-dose chemotherapy and the sequential administration of temsirolimus followed by high-dose chemotherapy in lung tumor growth inhibition. The tumor growth inhibition was significant in the first 3 weeks of treatment with temsirolimus. However, the tumor growth inhibition efficacies between the two chemotherapy regimens were similar (A). Effect of various treatments on tumor growth rate (B). The tumor growth inhibition was most significant after the mixed treatment (T + C + G) with a lower dose of chemotherapy, which is beneficial in reducing the cytotoxic effect. (∗p ≤ 0.05; ∗∗p ≤ 0.01). [file Presentation_1.zip › Supplementary Figure S1A.tif]

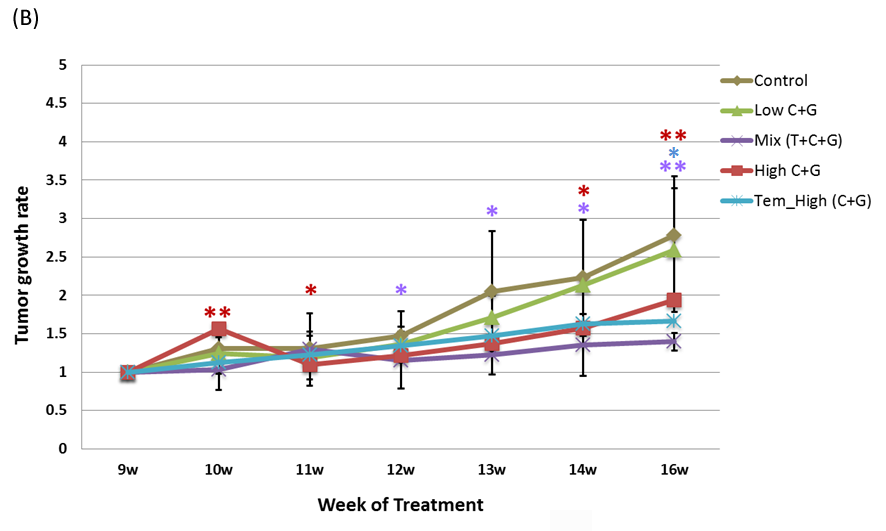

Supplement: FIGURE S1 — Effects of high-dose chemotherapy and the sequential administration of temsirolimus followed by high-dose chemotherapy in lung tumor growth inhibition. The tumor growth inhibition was significant in the first 3 weeks of treatment with temsirolimus. However, the tumor growth inhibition efficacies between the two chemotherapy regimens were similar (A). Effect of various treatments on tumor growth rate (B). The tumor growth inhibition was most significant after the mixed treatment (T + C + G) with a lower dose of chemotherapy, which is beneficial in reducing the cytotoxic effect. (∗p ≤ 0.05; ∗∗p ≤ 0.01). [file Presentation_1.zip › Supplementary Figure S1B.tif]

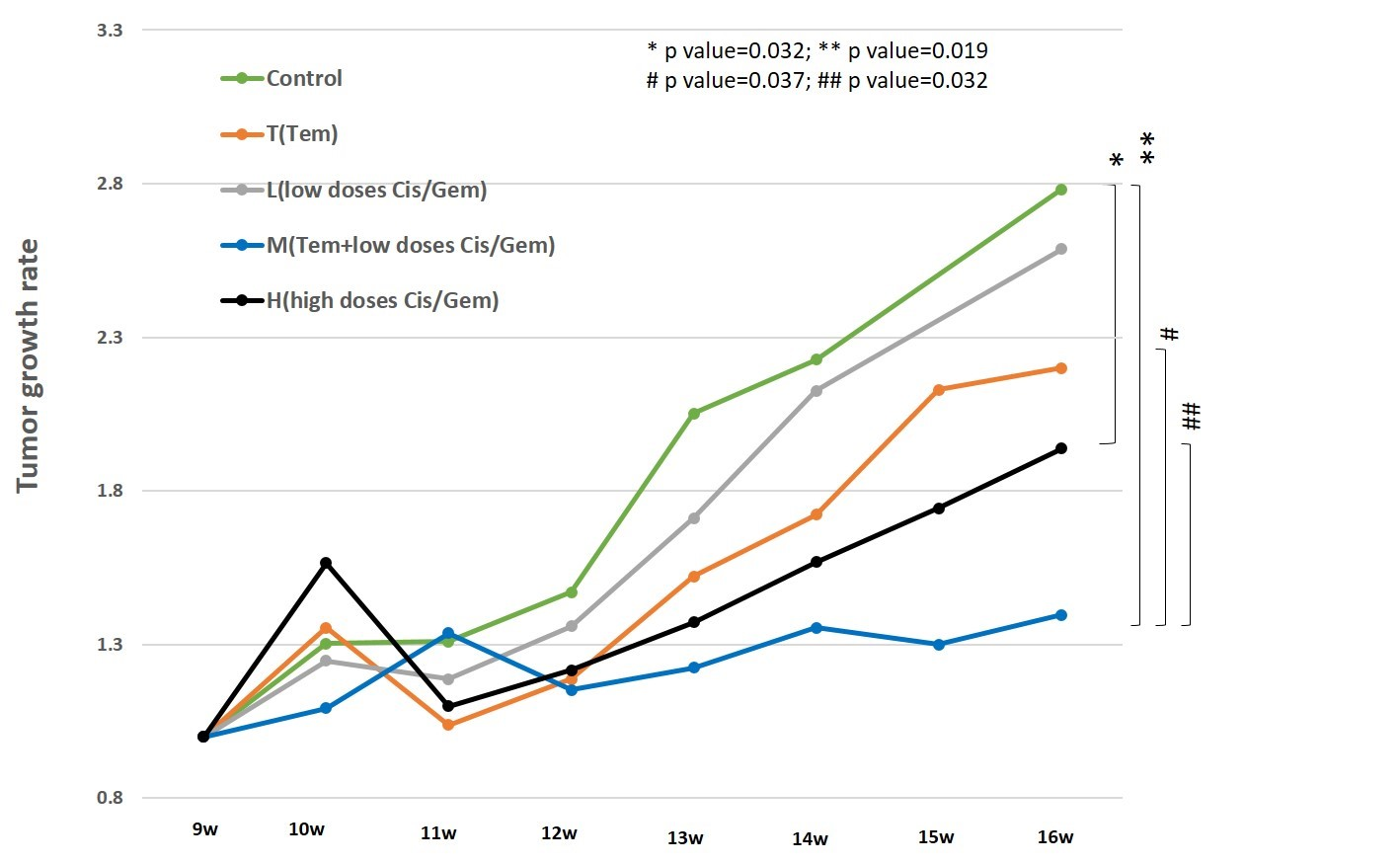

Supplement: FIGURE S1 — Effects of high-dose chemotherapy and the sequential administration of temsirolimus followed by high-dose chemotherapy in lung tumor growth inhibition. The tumor growth inhibition was significant in the first 3 weeks of treatment with temsirolimus. However, the tumor growth inhibition efficacies between the two chemotherapy regimens were similar (A). Effect of various treatments on tumor growth rate (B). The tumor growth inhibition was most significant after the mixed treatment (T + C + G) with a lower dose of chemotherapy, which is beneficial in reducing the cytotoxic effect. (∗p ≤ 0.05; ∗∗p ≤ 0.01). [file Presentation_1.zip › Supplementary Figure S2.tif]

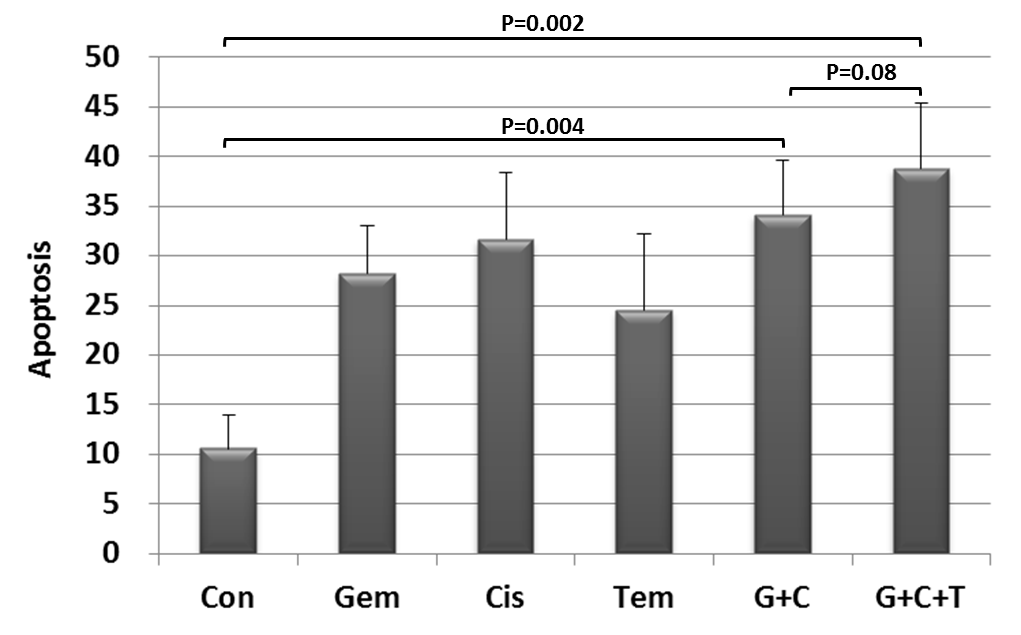

Supplement: FIGURE S1 — Effects of high-dose chemotherapy and the sequential administration of temsirolimus followed by high-dose chemotherapy in lung tumor growth inhibition. The tumor growth inhibition was significant in the first 3 weeks of treatment with temsirolimus. However, the tumor growth inhibition efficacies between the two chemotherapy regimens were similar (A). Effect of various treatments on tumor growth rate (B). The tumor growth inhibition was most significant after the mixed treatment (T + C + G) with a lower dose of chemotherapy, which is beneficial in reducing the cytotoxic effect. (∗p ≤ 0.05; ∗∗p ≤ 0.01). [file Presentation_1.zip › Supplementary Figure S3.tif]

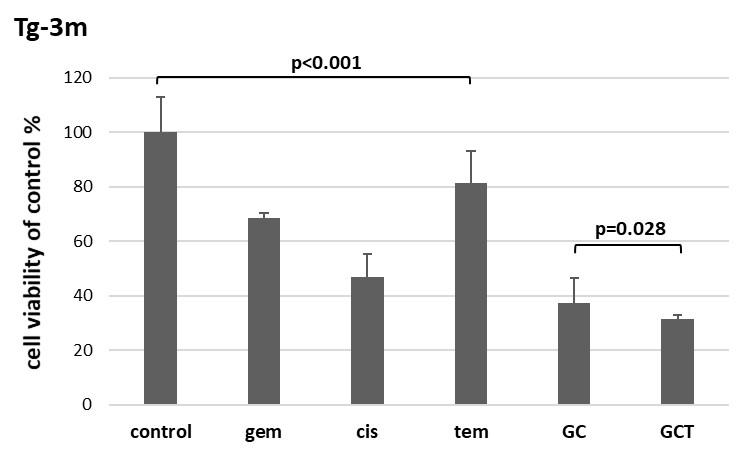

Supplement: FIGURE S1 — Effects of high-dose chemotherapy and the sequential administration of temsirolimus followed by high-dose chemotherapy in lung tumor growth inhibition. The tumor growth inhibition was significant in the first 3 weeks of treatment with temsirolimus. However, the tumor growth inhibition efficacies between the two chemotherapy regimens were similar (A). Effect of various treatments on tumor growth rate (B). The tumor growth inhibition was most significant after the mixed treatment (T + C + G) with a lower dose of chemotherapy, which is beneficial in reducing the cytotoxic effect. (∗p ≤ 0.05; ∗∗p ≤ 0.01). [file Presentation_1.zip › Supplementary Figure S4A.jpeg]

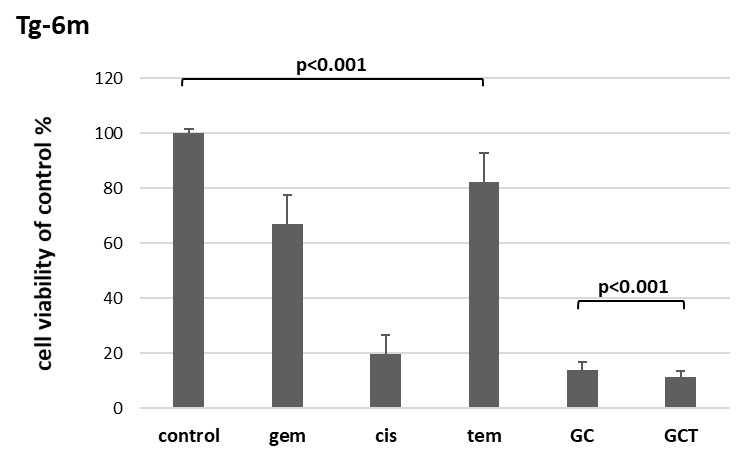

Supplement: FIGURE S1 — Effects of high-dose chemotherapy and the sequential administration of temsirolimus followed by high-dose chemotherapy in lung tumor growth inhibition. The tumor growth inhibition was significant in the first 3 weeks of treatment with temsirolimus. However, the tumor growth inhibition efficacies between the two chemotherapy regimens were similar (A). Effect of various treatments on tumor growth rate (B). The tumor growth inhibition was most significant after the mixed treatment (T + C + G) with a lower dose of chemotherapy, which is beneficial in reducing the cytotoxic effect. (∗p ≤ 0.05; ∗∗p ≤ 0.01). [file Presentation_1.zip › Supplementary Figure S4B.jpeg]

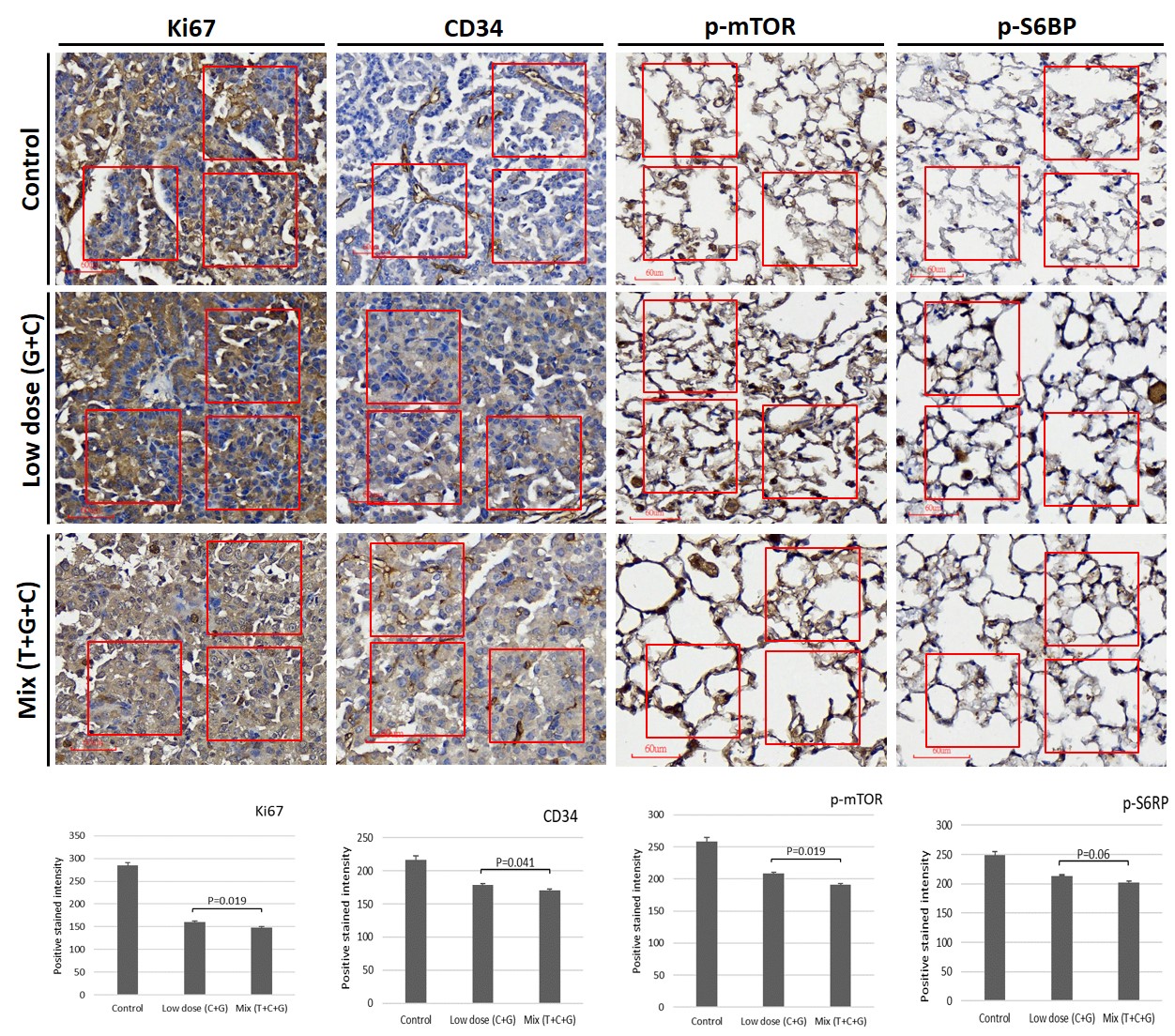

Supplement: FIGURE S1 — Effects of high-dose chemotherapy and the sequential administration of temsirolimus followed by high-dose chemotherapy in lung tumor growth inhibition. The tumor growth inhibition was significant in the first 3 weeks of treatment with temsirolimus. However, the tumor growth inhibition efficacies between the two chemotherapy regimens were similar (A). Effect of various treatments on tumor growth rate (B). The tumor growth inhibition was most significant after the mixed treatment (T + C + G) with a lower dose of chemotherapy, which is beneficial in reducing the cytotoxic effect. (∗p ≤ 0.05; ∗∗p ≤ 0.01). [file Presentation_1.zip › Supplementary Figure S5.jpg]
